# Tranexamic acid attenuates inflammatory response in cardiopulmonary bypass surgery through blockade of fibrinolysis: a case control study followed by a randomized double-blind controlled trial

**DOI:** 10.1186/cc6173

**Published:** 2007-11-07

**Authors:** Juan J Jimenez, Jose L Iribarren, Leonardo Lorente, Jose M Rodriguez, Domingo Hernandez, Ibrahim Nassar, Rosalia Perez, Maitane Brouard, Antonio Milena, Rafael Martinez, Maria L Mora

**Affiliations:** 1 Intensive Care Department, Hospital Universitario de Canarias, Ofra s/n La Cuesta, La Laguna, 38320, Spain; 2 Hematology Department, Hospital Universitario de Canarias, Ofra s/n La Cuesta, La Laguna, 38320, Spain; 3 Research Unit, Hospital Universitario de Canarias, Ofra s/n La Cuesta, La Laguna, 38320, Spain; 4 Cardiac Surgery Department, Hospital Universitario de Canarias, Ofra s/n La Cuesta, La Laguna, 38320, Spain; 5 Biochemistry and Central Laboratories, Hospital Universitario de Canarias, Ofra s/n La Cuesta, La Laguna, 38320, Spain

## Abstract

**Introduction:**

Extracorporeal circulation induces hemostatic alterations that lead to inflammatory response (IR) and postoperative bleeding. Tranexamic acid (TA) reduces fibrinolysis and blood loss after cardiopulmonary bypass (CPB). However, its effects on IR and vasoplegic shock (VS) are not well known and elucidating these effects was the main objective of this study.

**Methods:**

A case control study was carried out to determine factors associated with IR after CPB. Patients undergoing elective CPB surgery were randomly assigned to receive 2 g of TA or placebo (0.9% saline) before and after intervention. We performed an intention-to-treat analysis, comparing the incidence of IR and VS. We also analyzed several biological parameters related to inflammation, coagulation, and fibrinolysis systems. We used SPSS version 12.2 for statistical purposes.

**Results:**

In the case control study, 165 patients were studied, 20.6% fulfilled IR criteria, and the use of TA proved to be an independent protective variable (odds ratio 0.38, 95% confidence interval 0.18 to 0.81; *P *< 0.01). The clinical trial was interrupted. Fifty patients were randomly assigned to receive TA (24) or placebo (26). Incidence of IR was 17% in the TA group versus 42% in the placebo group (*P *= 0.047). In the TA group, we observed a significant reduction in the incidence of VS (*P *= 0.003), the use of norepinephrine (*P *= 0.029), and time on mechanical ventilation (*P = *0.018). These patients showed significantly lower D-dimer, plasminogen activator inhibitor 1, and creatine-kinase levels and a trend toward lower levels of soluble tumor necrosis factor receptor and interleukin-6 within the first 24 hours after CPB.

**Conclusion:**

The use of TA attenuates the development of IR and VS after CPB.

**Trial registration number:**

ISRCTN05718824.

## Introduction

Cardiopulmonary bypass (CPB) may activate an inflammatory response (IR) involving contact system, complement, cytokine, and coagulation-fibrinolytic cascades, among others. The coagulation-fibrinolytic cascades and the IR, though in many respects separate processes, are closely interconnected [1]. Several preoperative and perioperative risk factors for IR have been proposed [2,3]. The incidence of vasoplegic shock (VS), the most severe presentation of IR, may be as high as 10% [4].

Numerous strategies to reduce IR and bleeding in high-risk patients exist, among which is the use of aprotinin [5]. Like aprotinin, tranexamic acid (TA) inhibits fibrinolysis (that is, plasmin activity and D-dimer formation), but its effect on IR remains unclear. Additionally, there is evidence that fibrinolysis is a marker for the onset of systemic inflammation. [6].

This paper describes a study in two parts. First, we performed a case control study to determine risk factors associated with IR in patients who underwent CPB. Second, we carried out a randomized, double-blind, placebo-controlled study to test the hypothesis that inhibition of excessive fibrinolysis by TA could reduce the incidence of IR and VS after CPB. The second study was interrupted because of the high incidence of adverse effects observed in the placebo group. Thus, we present data of an interim analysis.

## Materials and methods

The study was approved by the institutional ethics committee of the University Hospital of the Canary Islands (La Laguna, Spain) and was conducted according to the Declaration of Helsinki. The study consisted of two parts.

### Part 1: Assessment of postoperative incidence and protective/risk factors for inflammatory response after cardiopulmonary bypass

After obtaining informed written consent, we prospectively enrolled 191 consecutive Caucasian adult patients scheduled for cardiac surgery with CPB between January 2002 and February 2003. To avoid the effect of confounding factors on the IR, patients with endocarditis and those admitted with cardiogenic shock or with intra-aortic counterpulsation balloon were excluded (*n *= 26). Finally, a total of 165 patients were included. No patients received perioperative anti-inflammatory agents such as corticosteroids or nonsteroidal anti-inflammatory drugs.

IR was clinically defined as a core body temperature of greater than 38°C (100.4°F) in the first 4 hours after intervention, a systemic vascular resistance index of less than 1,600 dyn-seconds/cm^5 ^per square meter, and a cardiac index of greater than 3.5 L/minute per square meter. VS was defined as persistent hypotension (mean arterial pressure of less than 70 mm Hg) requiring norepinephrine for at least 4 hours after failure to respond to appropriate volume expansion (pulmonary capillary wedge pressure of greater than 15 mm Hg). Serum concentrations of interleukin-6 (IL-6) were measured at 4 hours after CPB (Materials and methods, part 2). Risk factors associated with IR after CPB, including demographic variables, comorbid conditions, preoperative medication, duration of CPB, aortic crossclamp time, and the use of antifibrinolytic drugs, were investigated. Perioperative management of the groups was similar in the two studies (Materials and methods, part 2), except for the study medication. In this study, the surgeon decided when to use TA.

### Part 2: Prospective double-blind trial of tranexamic acid effect on inflammatory response after cardiopulmonary bypass

We performed a randomized, double-blind, placebo-controlled study with consecutive Caucasian adult patients undergoing elective CPB surgery from February to May 2004. Postoperative care of the patients was performed in a 24-bed intensive care unit (ICU) at a university hospital. We excluded emergency interventions, patients with a history of chronic coagulopathy (prothrombin time [PT] of less than 50% or international normalized ratio of greater than 2 and platelets of less than 50,000/mm^3 ^or aggregation dysfunction), renal failure (creatinine of greater than 2 mg/dL), chronic hepatopathy (Child B or higher degree), use of immunosuppressant drugs, endocarditis, sepsis in the first 24 hours after intervention, or unwillingness to enroll. Before CPB, participants had normal bleeding time, platelet collagen/epinephrine and collagen/ADP closure time, PT, activated partial thromboplastin time, and thrombin time. None of the patients received anti-inflammatory agents such as corticosteroids or nonsteroidal anti-inflammatory agents, including acetyl salicylate acid or clopidogrel or immunosuppressants, on the previous 5 days and the first 24 hours following intervention.

After informed written consent was obtained, patients were randomly assigned by independent pharmacists using a list of pseudorandomized numbers to receive coded infusions of either TA or placebo (0.9% saline) with doses of 2 g pre-CPB and post-CPB after protamine administration (using the same protocol as in the previous part of the study). The code was revealed once recruitment, data collection, and laboratory analyses were completed. The primary endpoint was to test the effect of TA on the incidence of IR and VS in patients undergoing elective CPB. Secondary endpoints were biological parameters related to inflammation, coagulation, and fibrinolysis systems.

### Data collection

Demographic variables, comorbid conditions, perioperative clinical data, and postoperative outcomes (IR, VS, duration of mechanical ventilation, postsurgical ICU stay and hospital stay, and mortality) were recorded. Core body temperature, biochemical determinations (hematology, inflammation, coagulation, and fibrinolysis), and hemodynamic parameters were recorded before intervention (baseline), on admission to the ICU after surgery (0 hours), and at 4 hours and 24 hours after intervention. In addition, blood loss measured by tube chest drainage and the amount of hemoderivatives used, as well as its frequency, were collected after intervention at the above time points and when chest tubes were removed (defined as total bleeding). Surgical risk was calculated by Parsonnet score.

Anesthetic procedures were standardized and consisted of an opioid-based anesthetic supplemented with volatile anesthetic and muscle relaxants. All interventions were performed by the same surgical team with wide experience in these surgical interventions. All patients were preoperatively monitored with a pulmonary artery continuous thermodilution catheter (Edwards Lifesciences LLC, Irvine, CA, USA). Neither heparin-coated circuits nor leukocyte filters were used. The extracorporeal circuit consisted of a hardshell membrane oxygenator (Optima XP; Cobe, Denver, CO, USA, or Quantum Lifestream International, Inc., Woodlands, TX, USA), a Tygon™ (Dideco s.r.l., Mirandola, Italy) extracorporeal circuit, and a Medtronic™ Biopump (Medtronic, Inc., Minneapolis, MN, USA) centrifugal pump. Below hypothermic temperatures of 28°C to 30°C, the pump flow was adjusted to maintain a mean arterial pressure of greater than 60 mm Hg and a flow index of 2.2 L/minute per square meter. Myocardial protection was achieved using antegrade, cold, St. Thomas 4:1 sanguineous cardioplegia. The circuit was primed with 30 mg of heparin followed by an initial dose of 3 mg/kg and further doses when necessary to achieve and maintain an activated clotting time of 480 seconds. To reverse the effect of heparin, protamine was used based on blood heparin levels measured by Hepcon^® ^(Medtronic, Inc.). A blood salvage device was used in all patients. The transfusion trigger was a hemoglobin threshold of less than 8 g/dL, PT of less than 50%, and platelets of less than 50,000/mm^3^. Fluid management was carried out to achieve 8 to 12 mm Hg of central venous pressure or 12 to 15 mm Hg of pulmonary artery occlusion pressure at zero positive end-expiratory pressure by infusions of crystalloids and colloids. Catecholamine support, when necessary, was used as follows: Norepinephrine was titrated to achieve a mean arterial pressure of greater or equal to 70 mm Hg, and dobutamine was titrated to achieve a cardiac index of greater or equal to2.5 L/minute per square meter. Amines were tapered off in steps of 0.02 and 1 μg/kg per minute, respectively.

### Cytokine levels

Soluble tumor necrosis factor receptor (STNFR)-1 and IL-6 (normal range: less than 5.9 pg/mL; intra-assay variation: 4.5%) were measured using an automatic immunoenzyme assay system (IMMULITE ONE™; Diagnostic Products Corporation, now part of Siemens AG, Munich, Germany). STNFR-1 EASIA (normal range: 3.4 to 10.8 ng/mL; intra-assay variation: 1.7%) are solid phase enzyme-amplified sensitivity immunoassays performed on a microtiter plate (, Biosource Technologies, Inc., Fleunes, Belgium).

### Coagulation and fibrinolysis determination

Quantitative plasminogen activator inhibitor 1 (PAI-1) antigen (normal range: 2 to 47 ng/mL; intra-assay variation: 3.7%) and tissue plasminogen activator antigen levels (normal range: less than 9.0 ng/mL; intra-assay variation: 4.2%) were measured using an enzyme-linked immunosorbent assay (IMUBIND^®^; American Diagnostica Inc., Stamford, CT, USA). D-dimer (normal range: less than 300 ng/mL; intra-assay variation: 3%) was measured using an immunoturbidimetric test (D-dimer PLUS; Dade Behring, now part of Siemens AG).

### Statistical analysis

Comparisons between groups (patients with and without IR or the TA group versus placebo group) were performed using the Pearson χ^2 ^test or Fisher exact test for categorical variables and the Student *t *test or the Mann-Whitney *U *test for continuous variables, as appropriate. Logistic regression analysis (forward stepwise conditional) was used to identify independent risk factors associated with IR. Initially, only variables with a *P *value of less than 0.15 (TA, clamping time, and mixed cardiac surgery) in the univariate analysis were incorporated. To perform the controlled study, a sample size of 100 patients was required to detect a statistically significant reduction of at least 20% in IR by TA. Assuming an incidence of 30% in the placebo group, a study population of 100 patients was expected to have 80% power to detect a 20% reduction in IR. For primary endpoint outcomes, all differences in preoperative variables with a *P *value of less than 0.15 in the univariate analysis of the controlled study were entered into a logistic regression analysis. Results for qualitative variables are expressed as frequency and percentage. Quantitative variables are expressed as mean ± standard deviation or as median and interquartile range in the case control study and as mean and 95% CI in the controlled study. A *P *value of less than 0.05 was considered statistically significant. For primary endpoint outcomes of the controlled study, exact *P *values are reported. SPSS version 12.2 (SPSS Inc., Chicago, IL, USA) was used.

## Results

### Part 1: Assessment of postoperative incidence and protective/risk factors for inflammatory response after cardiopulmonary bypass

**Figure 1:**
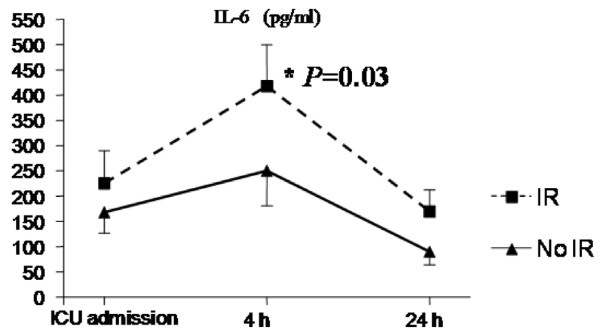
Levels of interleukin-6 (IL-6) at 4 hours between inflammatory response (IR) patients and non-IR patients. ICU, intensive care unit.

**Figure 2:**
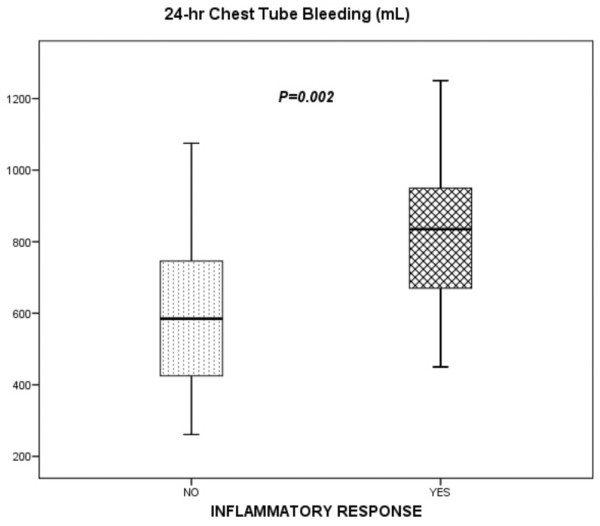
Relationship between 24-hour chest tube bleeding and inflammatory response. Horizontal lines represent the median, boxes encompass the 25th to 75th percentile, and error bars encompass the 10th to 90th percentile.

Of 165 patients, 34 (20.6%) fulfilled the criteria for IR. At 4 hours after intervention, patients who developed IR presented higher cardiac rates (107 ± 17 beats per minute [versus 87 ± 12 bpm; *P *< 0.001) and lower systolic arterial pressures (107 ± 20 mm Hg versus 136 ± 15.4 mm Hg; *P *< 0.001). These patients presented significantly higher levels of IL-6 at 4 hours: 418 ± 216 pg/mL versus 232 ± 198 pg/mL in the non-IR group (*P *= 0.033) (Figure 1). Also, IR patients showed significantly higher 24-hour postoperative bleeding of 835 (670 to 950) mL as compared to non-IR patients with 585 (425 to 746) mL (*P *= 0.002) with no significant differences in transfusion requirements between groups (Figure 2).

Table 1 shows demographic and clinical data of patients who developed IR as compared with those without IR. The only significant difference in the univariate analysis was the use of TA, which was associated with a lower incidence of IR (*P *= 0.002). IR was found in 26 (33%) of 79 patients who did not receive TA versus 8 (9%) of 86 patients who received TA. Initially, we included aortic clamping time (*P *= 0.11), mixed cardiac surgery (*P *= 0.05), and TA administration (*P *< 0.01). Only the use of TA proved to be an independent protective variable (odds ratio [OR] 0.38, 95% confidence interval [CI] 0.18 to 0.81; *P *= 0.009).

**Table 1:** Part 1. Patient characteristics and associations with inflammatory response after cardiopulmonary bypass

Variables	Inflammatory response (*n *= 34)	No inflammatory response (*n *= 131)	*P *value
Age, years	61 ± 12	61 ± 13	0.97
Gender			
Male, number (percentage)	24 (70)	88 (67)	0.70
Female, number (percentage)	10 (30)	43 (33)	0.70
Body mass index, kg/m^2^	28.5 ± 5	27.5 ± 4.2	0.20
Parsonnet score	13.6 ± 9.2	12.1 ± 6.8	0.35
Comorbidity			
Renal disease, number (percentage)	4 (11)	8 (6)	0.26
Diabetic status, number (percentage)	12 (35)	44 (34)	0.85
Angiotensin-converting enzyme inhibitors, number (percentage)	10 (29)	45 (34)	0.58
Cardiac intervention			
Coronary, number (percentage)	19 (56)	81 (62)	0.20
Valvular, number (percentage)	9 (26)	41 (31)	0.58
Both, number (percentage)	6 (17)	9 (7)	0.051
Reintervention, number (percentage)	2 (6)	6 (5)	0.75
Surgical data			
Total cardiopulmonary bypass time, minutes	101 ± 33	93 ± 33	0.20
Aortic clamping time, minutes	61.6 ± 27.3	54 ± 22.8	0.11
Tranexamic acid, number (percentage)	8 (26)	78 (60)	<0.01
No antifibrinolytics, number (percentage)	26 (76)	53 (40)	0.44
Intensive care unit stay, days	7.8 ± 6.4	3.2 ± 1.7	<0.01
Hospital stay, days	17.6 ± 20.5	9.1 ± 6.2	<0.01

Values are expressed as mean ± standard deviation.

Twenty (12%) of the 165 patients presented VS. In the non-TA group, 16 (20%) out of 79 patients developed VS. As expected, patients with IR were more likely to develop VS (58% versus 0%; *P *< 0.001). There were 3 deaths (1.8%) in the whole group; none of them had developed IR.

### Part 2: Prospective double-blind trial of tranexamic acid effect on inflammatory response after cardiopulmonary bypass

**Figure 3:**
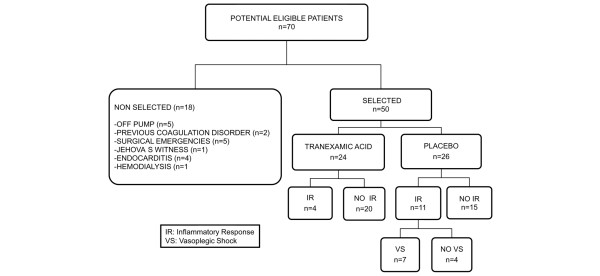
Randomized control trial flow diagram.

The study was interrupted by the ethics committee after the inclusion of 50 patients due to the higher proportion of severe bleeding observed in the placebo group during follow-up. The primary analysis was intention-to-treat and involved all patients who were randomly assigned. We studied 50 patients, 24 receiving TA and 26 placebo, from 68 consecutive patients, of whom 18 met criteria for exclusion (5 off-pump, 2 with previous surgery coagulation disorders, 5 surgical emergencies, 1 Jehovah's Witness, 4 with endocarditis, and 1 with chronic renal failure on hemodialisis) (Figure 3). Demographic variables, comorbidity, medical treatment, preoperative biochemical data, and surgical procedures were similar in the two groups (Table 2).

**Table 2:** Part 2. Baseline clinical data of controlled study (*n *= 50)

Variables	Tranexamic acid (*n *= 24)	Placebo (*n *= 26)	*P *value
Demographic			
Age, years	66 (63–70)	67 (62–71)	0.91
Male gender, number (percentage)	12 (50)	15 (57)	0.58
Body mass index, kg/m^2^	28 (25.8–30.1)	28.1 (36.4–29.7)	0.98
Parsonnet score	13.1 (11.8–15.5)	17.5 (13.7–21.3)	0.07
Comorbidity			
Cardiopathy, number (percentage)			
Coronary	13 (54)	12 (46)	0.71
Valve	9 (38)	10 (39)	0.68
Mixed	2 (8)	4 (15)	0.44
Medical treatment			
Angiotensin-converting enzyme inhibitors, number (percentage)	11 (61)	7 (39)	0.16
Calcium channel blockers, number (percentage)	6 (60)	4 (40)	0.39
Preoperative parameters			
Platelet count, × 10^3^/mL	210 (186–234)	210 (186–239)	0.68
Hemoglobin, g/dL	14.1 (13.5–14.6)	13.6 (12.8–14.4)	0.42
International normalized ratio	1.08 (1.05–1.12)	1.09 (1.05–1.14)	0.97
D-dimer, ng/mL	250 (166–333)	275 (215–325)	0.34
Plasminogen activator inhibitor 1, ng/mL	34.2 (29–39.5)	35.2 (29.4–41.1)	0.95
Surgical data			
Cardiopulmonary bypass time, minutes	82 (71–94)	85 (74–96)	0.30
Aortic clamping time, minutes	51 (44–58)	55 (47–62)	0.35
Temperature after cardiopulmonary bypass, degrees Celsius	35.3 (34.9–35.6)	35.1 (34.7–35.3)	0.24
Total heparin dose, UI/kg	430 (400–470)	420 (400–440)	0.69
Total protamine dose, mg/kg	2.7 (2.5–3)	2.7 (2.6–2.9)	0.72
Blood salvage device, mL	681 (605–756)	764 (694–833)	0.12

Values are expressed as mean and 95% confidence interval or as frequency and percentage.

The incidence of IR was significantly lower in the TA group (17%) than in the placebo group (42%) (*P *= 0.047). TA showed a protective effect for IR (OR 0.1, 95% CI 0.01 to 0.7) after adjusting for Parsonnet score, aortic clamping time, and type of surgery. As compared with the TA group, the relative risk for developing IR was 2.47 for the placebo group (97.5% CI 1.1 to 5.7). The absolute risk difference was 25%. Thus, the number needed to treat to reduce IR was 4 patients (97.5% CI 2 to 20 patients). The incidence of VS was 0% in the TA group versus 23% in the placebo group (*P *< 0.001).

**Figure 4:**
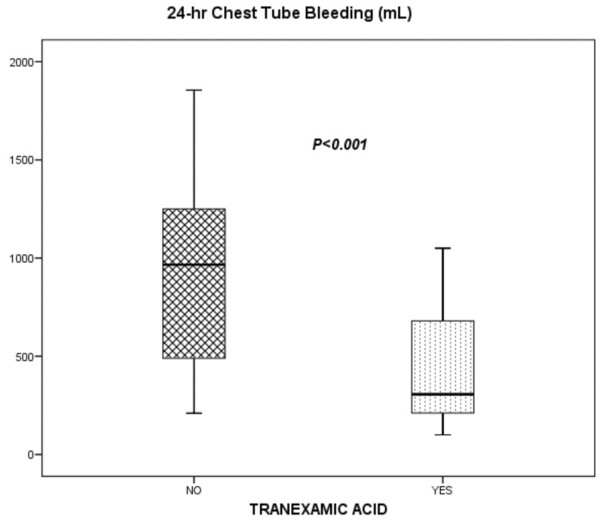
Twenty-four-hour chest tube bleeding between tranexamic acid and placebo groups. Horizontal lines represent the median, boxes encompass the 25th to 75th percentile, and error bars encompass the 10th to 90th percentile.

The TA group had significantly lower 24-hour chest tube bleeding (*P *< 0.001) (Figure 4) and transfusion requirements before ICU discharge compared with the placebo group. In addition, the TA group required significantly less vasopressor medication and mechanical ventilation time. We did not find significant differences in duration of ICU stay or hospital stay after surgery between groups (Table 3). One patient from the placebo group required reintervention due to nonsurgical bleeding. There were no deaths in this study.

**Table 3:** Part 2. Clinical outcomes of the controlled study

Variables	Tranexamic acid (*n *= 24)	Placebo (*n *= 26)	*P *value
D-dimer at 0 hours, ng/mL^a^	448 (270–625)	1,069 (951–1,189)	<0.01
D-dimer at 4 hours, ng/mL	499 (364–633)	974 (880–1,069)	<0.01
D-dimer at 24 hours, ng/mL	433 (280–608)	976 (868–1,064)	<0.01
Plasminogen activator inhibitor 1 at 4 hours, ng/mL	88 (35–140)	129 (70–188)	0.04
Creatine-kinase at 0 hours, U/L	261 (199–322)	327 (270–383)	0.03
Creatine-kinase MB at 0 hours, U/L	41 (35–47)	63 (50–77)	<0.01
STNFR-1 at 4 hours, ng/mL	1,274 (958–1,590)	1,656 (1,175–2,138)	0.09
Interleukin-6 at 4 hours, pg/mL	236 (140–332)	362 (250–474)	0.12
Interleukin-6 at 24 hours, pg/mL	87 (61–114)	119 (88–151)	0.07
Twenty-four-hour bleeding, mL	464 (308–620)	1,037 (771–1,303)	<0.01
Total bleeding, mL	835 (407–1,263)	1,466 (1,116–1,818)	<0.01
RBC^b ^units transfused within the first 4 hours (percentage)^c^	1 (4)	2 (7)	0.39
RBC^b ^units until chest tube withdrawal (percentage)^c^	9 (38)	19 (73)	0.01
Plasma units until chest tube withdrawal (percentage)^c^	1 (4)	8 (31)	0.02
Inflammatory response after CPB, number (percentage)	4 (17)	11 (42)	0.047
Vasoplegic shock, number (percentage)	0	7 (27)	<0.01
Norepinephrine, hours	1.2 (0.5–2.4)	25.4 (5.6–45)	0.02
Mechanical ventilation, hours^d^	6.5 (5–13.5)	12 (7–24)	0.02
Intensive care unit stay, days^d^	3 (2–5.5)	3.5 (2–5)	0.96
Postsurgical hospital stay, days^d^	4.5 (3–6)	4 (2–5)	0.34

Values are expressed as mean and 95% confidence interval or as frequency and percentage. ^a^0 hours represents intensive care unit admission after cardiopulmonary bypass (CPB); ^b^total red blood cell (RBC) and plasma until chest tube withdrawal; ^c^percentage of transfused patients; ^d^values are expressed as median and interquartile range. STNFR-1, soluble tumor necrosis factor receptor type 1.

**Figure 5:**
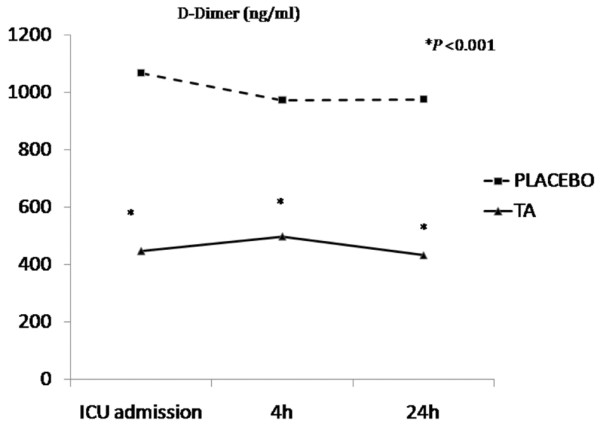
Differences between tranexamic acid (TA) (solid line) and placebo (dotted line) in D-dimer levels. ICU, intensive care unit.

Table 3 shows the biological variables studied in both groups. Significantly lower D-dimer (Figure 5), PAI-1, and creatine-kinase levels were observed in patients in the TA group within the first 24 hours after CPB; lower levels of STNFR and IL-6 were observed in the TA group, but these differences were not significant. The remaining variables (coagulation parameters) did not show significant differences (data not shown).

## Discussion

### Part 1: Assessment of postoperative incidence and protective/risk factor for inflammatory response after cardiopulmonary bypass

According to previous reports, it is widely accepted that a systemic response is induced in nearly all patients undergoing open-heart surgery [1]. The occurrence rate of a hyperdynamic state after CPB has been reported to be as low as 4%. [7] and as high as 44% [8]. Indeed, much of the difference in prevalence may relate to the criteria used to define the vasodilatory syndrome [9]. The American College of Chest Physicians/Society of Critical Care Medicine consensus proposed a very sensitive, but very low-specificity, definition for systemic IR syndrome [10]. This definition is often inappropriate for cardiac surgery patients (mechanically ventilated, hypothermic, with pacemakers, and so on), and therefore we applied a definition based on hemodynamic data provided by the latest International Definitions Conference [11]. Other studies have proposed definitions based on analytical data such as high levels of IL-6 [12], whose serum concentrations correlate with morbidity and mortality following pediatric cardiac surgery [13]. The present study has shown that patients who fulfilled clinical criteria also had higher levels of IL-6. Therefore, the definition used seemed to be suitable to identify protective or risk factors for IR after CPB, even though this clinical picture may vary from mild to severe form. IR was found in one fourth of the patients, of whom more than half developed VS. TA was significantly associated with a lower incidence of IR. The incidence in those patients who did not receive TA was nearly one third, similar to other reports [12]. Thus, the next step was to test this hypothesis using an experimental design.

### Part 2: Prospective double-blind trial of tranexamic acid effect on inflammatory response after cardiopulmonary bypass

The trial was interrupted by the ethics committee due to the adverse effects (excessive bleeding) observed in the placebo group during follow-up. Our results indicate that TA reduces the incidence of IR and VS in CPB patients as well as postoperative bleeding and hemoderivative requirements. Several mechanisms have been proposed to explain the development of IR after CPB, such as contact activation, ischemia-reperfusion, and endotoxemia. These initiating factors may activate numerous systems involving complement, cytokines, immune cellular response with dysfunction of endothelium, and alteration of coagulation-fibrinolytic cascades [1]. This activation exposes patients to either immediate risk of major bleeding [14] or IR, as we saw in the first part of the study. The IR in cardiac surgery is closely related to hemostatic alterations. [15]. In this sense, higher D-dimer and IL-6 levels have been found in CPB patients with vasoplegic syndrome. [16]. In fact, IR and major bleeding could be considered as final outcomes of the same triggering stimulus, so that hyperfibrinolysis could play an important role in these processes. [17,18]. The suppression of excessive plasmin activity or D-dimer formation may play an important role in the generation of proinflammatory cytokine (IL-6) during and after CPB [5], which has been reported to be involved in circulatory dysregulation and metabolic derangement [4].

TA, an antifibrinolytic agent. [19], reduces bleeding and transfusion requirements after cardiac surgery. [20,21]. A synthetic derivative of the amino acid lysine, TA exerts its antifibrinolytic effect through the reversible blockade of lysine-binding sites on plasminogen molecules. However, the effect of TA on IR during cardiac surgery and CPB has received little attention [22]. In our study, low levels of D-dimer at all postoperative time points in the TA group clearly suggest that these patients experienced less secondary fibrinolysis which leads to reduced postoperative bleeding. Lower levels of PAI-1 at 4 hours may reflect less previous activation of fibrinolysis with less secondary production. We observed no striking changes in coagulation and complement parameters in the TA group. However, STNFR levels and IL-6 levels at 4 hours, which have been implicated in the development of postoperative morbidity after CPB [23], were lower, as were myocardial enzymes on admission, which may reflect a reduced IR [24] and thus less perioperative insult. Casati and colleagues [25] have proven that TA can effectively decrease postoperative IL-6 levels in this context. Blood transfusions are able to alter the IR, including cytokine concentrations of IL-6. However, we suppose that an influence of transfusions on the postoperative development of IR can be ruled out by the fact that only three patients were transfused before setting up the clinical criteria for IR. Furthermore, the number of red blood cell units given during the first hours of the postoperative period did not differ significantly between groups. Finally, due to the fact that vasodilator drugs may interact with vascular resistance, the inclusion of temperature as part of the clinical criteria rules out the confounding effect of these drugs.

The TA patients needed smaller amounts of vasopressors and shorter duration of mechanical ventilation. Greater bleeding may lead to higher doses of vasopressor but not simply because of a direct mechanistic principle. Other factors are implicated; there is evidence that several shared key components of IR are activated in major bleeding [26] and in vasoplegia after CPB. [16]. Therefore, we may consider that the use of a vasopressor does not depend exclusively on the amount of bleeding. We believe that TA could attenuate inflammatory changes through blockade of fibrinolysis and may modulate interactions between the different systems involved in the global response to CPB [1].

### Limitations of the study

Even though greater postoperative bleeding was associated with IR after CPB, a limitation was the failure to determine fibrinolysis parameters in the first part of the study. The main limitation of part 2 of the study is the sample size. However, this was a randomized controlled study and baseline data were comparable between groups. Additionally, although inclusion of patients was prematurely stopped, data analysis demonstrated that TA attenuates IR in patients after CPB. This small sample size could lead to a type II error regarding secondary endpoints, such as durations of hospital stay and ICU stay.

## Conclusion

The use of TA attenuates the development of IR and VS after CPB, with hyperfibrinolysis playing a predominant role in their development.

## Key messages

• Hyperfibrinolysis may play a role in inflammatory response (IR) after cardiopulmonary bypass (CPB).

• Inhibition of fibrinolysis with tranexamic acid may attenuate IR after CPB.

## Abbreviations

CI = confidence interval; CPB = cardiopulmonary bypass; ICU = intensive care unit; IL-6 = interleukin-6; IR = inflammatory response; OR = odds ratio; PAI-1 = plasminogen activator inhibitor 1; PT = prothrombin time; STNFR = soluble tumor necrosis factor receptor; TA = tranexamic acid; VS = vasoplegic shock.

## Competing interests

The authors declare that they have no competing interests.

## Authors' contributions

JJJ and JLI were responsible for the study design, data collection, processing blood samples during the study, statistical analysis, data interpretation, and drafting the manuscript. LL, RP, MB, and MLM were responsible for data collection and processing blood simples during the study and provided useful suggestions. JMR was responsible for determination of coagulation-fibrinolysis parameters and interpretation. IN and RM were the surgical team and were responsible for preoperative clinical and analytical data collection. AM was responsible for the determination of complement, leptins, soluble tumor necrosis factor receptors, interleukin-6, and interpretation. DH was responsible for the statistical analysis, data interpretation, and drafting the manuscript. All authors read and approved the final manuscript.
